# On Hemiplegia

**Published:** 1894-12-29

**Authors:** 


### ON HEMIPLEGIA.

Among the more common varieties of disease depending on organic changes in the nervous system, hemiplegia occupies a prominent place. This affection is characterised by more or less complete paralysis of the arm and leg on the same side of the body, and depends in the majority of instances on a lesion on the opposite side of the brain.

The onset of the paralysis is frequently sudden, and associated with a more or less prolonged loss of consciousness. The degree of suddenness of the onset of the symptoms affords a clue to the nature of the lesion, being most rapid when due to embolism, and most gradual when the result of thrombosis while hæmorrhage occupies an intermediate position as regards time. The patient may exhibit various convulsive movements, notably that of turning his head and eyes to one side—the side opposite to that on which he is paralysed. On recovering consciousness it will be found that the whole of one side is to some extent enfeebled. The left side is the most frequently affected in those cases which arise from embolism, and fortunate it is that this is so, for in paralysis of the right side of the body there is frequently a loss of the power of speech which is often persistent.

In hemiplegia of the type we have been considering there is no very appreciable interference with sensation, but in the much rarer condition depending on a lesion in the spinal cord there is loss of sensation on the opposite side of the body to that on which the motor paralysis exists.

The paralysed limbs may become rigid at an early period of the disease, but this is not so common as the occurence of later contractures which are apt to remain to a greater or less degree, all the joints being bent and stiffened.

Early rigidity occurs in some meningeal hæmorrhages, or when the blood has broken into the ventricles. The slighter attacks, however, are more favourable, and the limbs gradually recover power, the leg in the majority of instances recovering before the arm. This recovery of motor power is considerably aided by the application of electricity and regular massage of the muscles, each joint being also subjected to passive movements daily. These measures prevent the wasting of the paralysed muscles, which would otherwise take place from the effects of disuse, and also diminish the tendency to the more formidable later contracture and deformity. The patient is often soon able to get about with a little assistance, such as a stick, and should be carefully warned of the danger of tripping, as the toes tend to drag along the ground on lifting the foot, giving a most characteristic gait. Hemiplegia is not common in early life, but when it occurs it is apt to lead to imperfect growth of the affected side, and occasionally irregular movements in the muscles of the limbs, thus seriously disabling the patient.

